# Characterization of BAT activity in rats using invasive and non-invasive techniques

**DOI:** 10.1371/journal.pone.0215852

**Published:** 2019-05-15

**Authors:** Andreas Paulus, Petronella A. van Ewijk, Emmani B. M. Nascimento, Marijke De Saint-Hubert, Geert Hendrikx, Andrea Vogg, Ivo Pooters, Melanie Schnijderberg, Joris Vanderlocht, Gerard Bos, Boudewijn Brans, Vera B. Schrauwen-Hinderling, Felix M. Mottaghy, Matthias Bauwens

**Affiliations:** 1 Department of Radiology and Nuclear Medicine, Maastricht University Medical Center (MUMC+), Maastricht, The Netherlands; 2 NUTRIM School of Nutrition and Translational Research in Metabolism, Department of Nutrition and Movement Sciences, Maastricht University, Maastricht, The Netherlands; 3 Department of Nuclear Medicine, University Hospital RWTH Aachen University, Aachen, Germany; 4 CARIM, Maastricht University, Maastricht, Netherlands; 5 Hematology, Department of Internal Medicine, School of Oncology and Developmental Biology, Maastricht University Medical Center, Maastricht, The Netherlands; 6 Central Diagnostic Laboratory, Maastricht University Medical Center, Maastricht, The Netherlands; Institute of Zoology, CHINA

## Abstract

**Introduction:**

Brown adipose tissue (BAT) is considered as a potential target for combating obesity in humans where active BAT metabolizes glucose and fatty acids as fuel resulting in heat production. Prospective studies in humans have been set up to further study the presence and metabolic activity of BAT mostly using Positron Emission Tomography (PET) imaging in cold-stimulated conditions with the radiolabeled glucose derivative [^18^F]FDG. However, radiotracers beyond [^18^F]FDG have been proposed to investigate BAT activity, targeting various aspects of BAT metabolism. It remains questionable which tracer is best suited to detect metabolic BAT activity and to what extent those results correlate with *ex vivo* metabolic BAT activity.

**Methods:**

PET and Single Photon Emission Computed Tomography (SPECT) imaging, targeting different aspects of BAT activation such as glucose metabolism, fatty acid metabolism, noradrenergic stimulation, blood perfusion and amino acid transport system, was performed immediately after injection of the tracer in rats under different temperatures: room temperature, acute cold (4 ⁰C for 4 h) or acclimated to cold (4 ⁰C for 6 h per day during 28 days). Furthermore, Magnetic Resonance Spectroscopy (MRS)-derived BAT temperature was measured in control and cold-acclimated rats.

**Results:**

At room temperature, only [^18^F]FDG visualized BAT. Glucose metabolism, fatty acid metabolism, noradrenergic stimulation and blood perfusion showed a clear tracer-dependent twofold increase in BAT uptake upon cold exposure. Only the tracer for the amino acid transport system did not show BAT specific uptake under any of the experimental conditions. MRS demonstrated that cold-acclimated animals had BAT with a stronger heat-production compared to control animals.

**Conclusion:**

BAT activity following cold exposure in rats was visualized by several tracers, while only [^18^F]FDG was also able to show BAT activity under non-stimulated conditions (room temperature). The variances in uptake of the different tracers should be taken into account when developing future clinical applications in humans.

## Introduction

Brown adipose tissue (BAT) has gained considerable attention over the last decade, as its appearance and function in humans is becoming more elucidated. The main function of BAT is to dissipate energy in the form of heat, a process mediated by the mitochondrial uncoupling protein 1 (UCP1), in response to cold exposure [1, 2]. BAT is highly vascularized, densely innervated by the sympathetic nervous system and has thermogenic capacity that can significantly influence homeostasis [3–5].

The presence and/or activity of BAT can be non-invasively visualized in humans using magnetic resonance imaging (MRI), computed tomography (CT), thermography and molecular imaging [6–9]. Thermography is a relatively cheap technique, able to quantify elevations in skin temperature resulting from increased BAT activity [9, 10]. Since skin temperature is only indirectly correlated to BAT abundance (R^2^ < 0.3) [10], this technique is not optimally suited for directly monitoring BAT activity. MRI and CT are capable of visualizing soft tissue with a high spatial resolution but struggle to distinguish between white adipose tissue (WAT) and BAT [11]. These techniques quantify the decrease in fat content during cold exposure as a reflection of BAT activation. Molecular imaging, using either positron emission tomography (PET) or single photon emission computed tomography (SPECT) is performed with different radiopharmaceuticals. In this study we employed 2-[^18^F]fluorodeoxyglucose ([^18^F]FDG), 14-[^18^F]fluoro-6-thia-heptadecanoic acid ([^18^F]FTHA), [^99m^Tc]Tc-2-methoxy-isobutyl-isonitrile ([^99m^Tc]TcMIBI) and [^123^I]-metaiodobenzylguanidine ([^123^I]MIBG). These radiopharmaceuticals are able to visualize and quantify BAT activity in humans [12–17]. [^18^F]FDG mainly visualizes glucose transport and is the most frequently used tracer to image BAT due to its high availability. However fatty acids are the main metabolized substance class in BAT [18, 19] and therefore [^18^F]FDG might largely underestimate BAT activity. In addition [^18^F]FDG BAT uptake is verifiably reduced in diabetic patients [20] due to their insulin resistance. [^18^F]FTHA visualizes uptake of free fatty acids (probably mainly via CD36 [21]). Fatty acids are the main metabolized substance class in BAT and might therefore be a good measure for BAT activation state. [^123^I]MIBG, a false neurotransmitter analog of norepinephrine, visualizes the density of sympathetic nerve endings as it is taken up, concentrated and not further catabolized by the presynaptic nerve terminal [22]. Norepinephrine, which is released by the sympathetic nervous system is a known activator for BAT [23, 24] and therefore imaging with [^123^I]MIBG is a good indication of the susceptibility of BAT to be activated. [^99m^Tc]TcMIBI visualizes perfusion of tissue by binding to mitochondria rich cells. BAT cells have a high number of mitochondria and in previous studies it was shown that [^99m^Tc]TcMIBI is able to visualize BAT under basal conditions [12, 13]. In acute cold conditions uptake was only slightly pronounced and it was speculated that [^99m^Tc]TcMIBI only shows increased uptake after cold acclimation. Additionally [^123^I]I-Phenylalanine ([^123^I]IPA) (targeting LAT1-4 amino acid transport system density) was used. [^123^I]IPA has never been reported to visualize BAT clinically or preclinically, but as it correlates to a key amino acid transport system we feel it is important to include it in our study.

PET and SPECT images indicating presumed regions of BAT can also be selectively identified on CT and MRI images, and mRNA and protein analysis from tissue samples from these regions confirmed BAT-characteristics [25, 26]. It has also been shown that [^18^F]FDG uptake on PET images correlates positively to cold outside temperatures and negatively to a subjects BMI, in correspondence with predictions from in vitro work [27]. However, ex vivo and in vitro data can vary greatly within a specific tissue or type of cell culture [28], and it remains an open question just to what extent intra-tissue measurements from each of these techniques truly represents its metabolic activity.

The most straightforward method to activate BAT *in vivo* is exposure to acute cold but also cold acclimation further stimulates [^18^F]FDG PET uptake in humans [6, 29, 30]. However no other radiopharmaceuticals have been used in a clinical setting to evaluate the effect of cold acclimation, although Baba *et al*. compared a number of tracers ([^201^Tl]thaliumchloride, [^123^I]MIBG, ([^99m^Tc]TcMIBI, [^18^F]- or [^3^H]FDG, [^3^H]-l-methionine, and [^3^H]thymidine) to assess BAT uptake in rodents after acute cold exposure [12].

Therefore 3 questions remain: 1) which aspects of BAT metabolism can best be used to investigate BAT activity upon acute cold exposure and cold acclimation? 2) which radiopharmaceutical is most suitable to evaluate this aspects? 3) to what extent does data acquired using radiopharmaceuticals correlate with ex vivo findings and data acquired by other modalities such as MR Spectroscopy (MRS)?

In our study, we primarily investigated to what extent different radiopharmaceuticals are suitable to quantify response of BAT to acute cold and cold acclimation in rats. For this reason, we used a rat model which was acutely exposed to cold or acclimated to cold. We compared tracers that were previously described to visualize BAT in clinical setting, namely [^18^F]FDG, [^18^F]FTHA, [^123^I]MIBG, [^99m^Tc]TcMIBI and ([^123^I]IPA). Furthermore, using MRS, we visualized and quantified BAT in rat following acute cold or cold acclimation by means of gradient-echo sequence (Fast Low Angle Shot (FLASH)).

## Materials & methods

### Animal model

**Fig 1:**
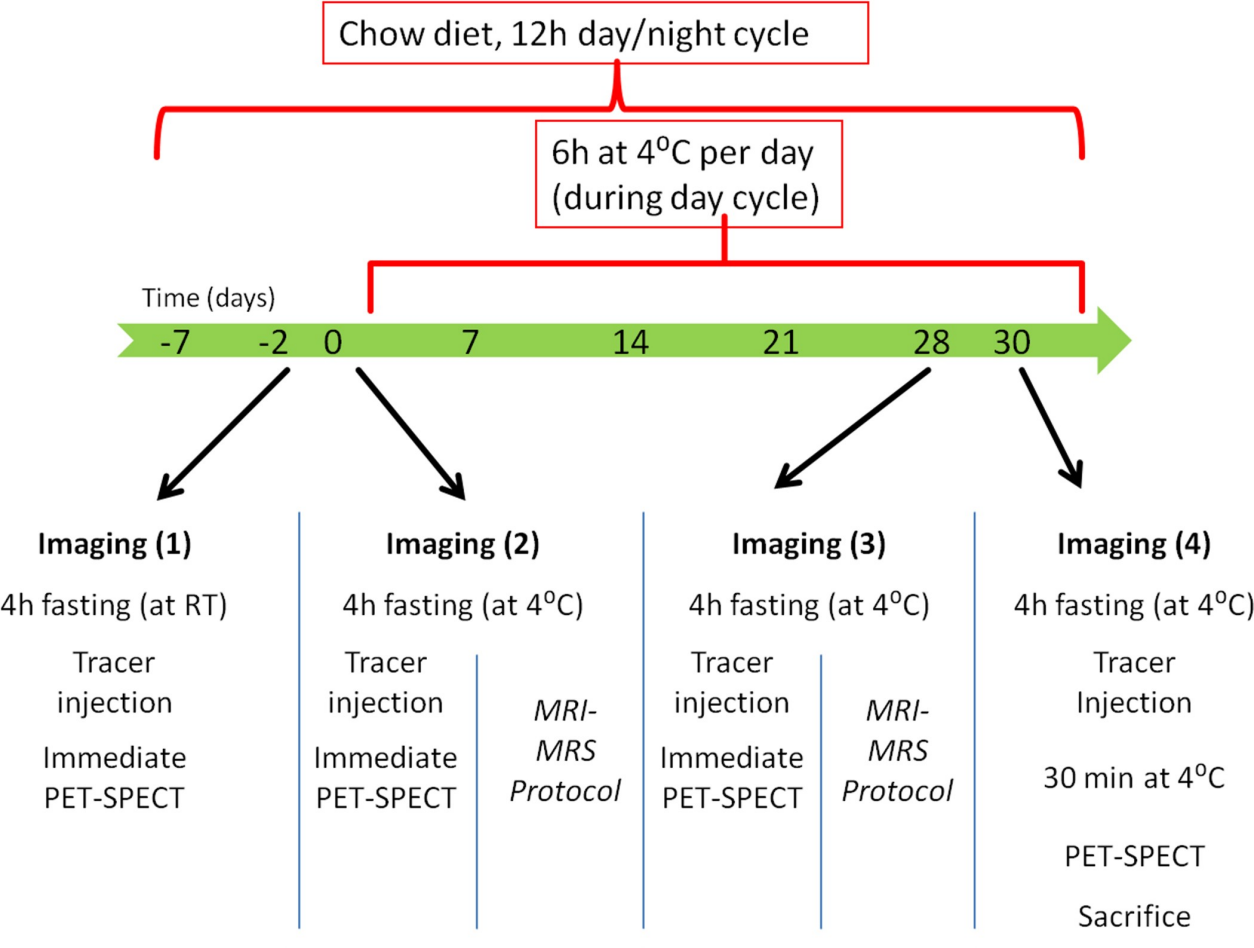
Study design. Imaging with each tracer was performed sequentially in baseline conditions (day -2), after acute cold exposure (day 0), after cold acclimation (day 28) and after tracer distribution in the cold (day 30). Additionally MRI/MRS was performed with another groupe at day 0 and day 28.

All animal studies were approved by the local animal ethical committee of the Maastricht University; with the internal permit number: DEC 2012–001. Male 12-week old Wistar rats were acquired from Harlan and housed under controlled temperatures of 22°C ± 1°C and 55–75% air humidity, in a 12 h light–dark cycle with water and food chow ad libitum. 36 animals were, after an initial week of normal housing, placed at 4 ⁰C for 6 h per day (9 am-3 pm) (food/drink ad libitum) during 28 days (Fig 1). At day -2 animals were scanned with one of the listed radiotracer to determine the control (room temperature) condition. To measure the acute cold conditions animals were scanned at day 0 after 4 h cold exposure and cold acclimated conditions were obtained after 28 days of cold exposure for 6 h/day. At day 30 cold exposed animals were injected in the cold with the PET/SPECT tracer and were further exposed to cold for 30 min before they were scanned to see if cold exposure during injection has an effect. 8 animals served as controls and were maintained at room temperature throughout the experiment. The physiological impact of the experiment was assessed by comparing food uptake, weight gain and discomfort in cold-acclimated and room temperature groups of animals. In addition, interscapular adipose tissue (iAT) was dissected, divided into BAT and WAT and weighed to determine its volume in sacrificed animals.

### Tracers: Radiosynthesis and formulation

[^18^F]FDG was purchased from GE Healthcare. Sestamibi (MIBI) was labeled with technetium-99m according to manufacturer guidelines (Mallincrodt Medical). Non-carrier added [^123^I]MIBG was prepared as suggested by the manufacturer of the precursor [31]. In short, synthesis was achieved by reacting iodine-123 with a polystyrene resin of dibutylstannyl benzylguanidine (Molecular Insight) in an oxidizing solution, transferring the [^123^I]MIBG over a cation exchange filter, rinsing with saline solution and finally collecting the non-carrier-added tracer by a phosphoric acid/ascorbic acid solution. After neutralization with NaOH a final volume of about 2ml is reached and the solution is ready for injection. The synthesis of ([^123^I]IPA) was adapted from earlier publications [32, 33]. Non-carrier added [^18^F]FTHA was prepared according to an adapted procedure from [34], starting from benzyl-14-(R,S)-tosyloxy-6-thiaheptadecanoate (ABX) as a precursor. The final purified compound was formulated using a 0.1% rat serum albumin solution to ensure solubility. All tracers showed a radiochemical purity of at least 98%.

### Imaging–general and anesthesia

PET, SPECT and MRI was performed according to the timeline represented in Fig 1. The animals were divided into subgroups, with group A, B, C, D and E (n = 4 or more per group) being scanned with respectively [^18^F]FDG, [^18^F]FTHA, [^123^I]MIBG, [^99m^Tc]TcMIBI and [^123^I]IPA and group F was used for MRI (n = 8). Imaging started at 1 pm. Prior to any scan or animal sacrifice, animals were fasted for 4 hours at either room temperature (baseline control conditions) or at 4 ⁰C (exposure to cold). PET/SPECT imaging was performed under pentobarbital anesthesia (0.1 ml of a 60 mg/ml solution per 100 g body weight, i.p.), as this sedative was reported to show the least side effects on BAT activity[35]. MRI/MRS imaging, due to its study duration (2.5–3 h per animal) and difficulty to access the animal, did not allow pentobarbital, so isoflurane was used (O_2_ as carrier, 3% for initial sedation, 1.8% for maintenance).

### Imaging (PET, SPECT) and biodistribution

Animals were sedated and subsequently injected in a tail vein under the camera (μPET Focus 120, Siemens, with a 1.4 mm spatial resolution or U-SPECT, MiLabs, with a 0.6 mm resolution) with 20-50MBq [^18^F]FDG (group A), 20–50 MBq [^18^F]FTHA (group B), 100MBq [^123^I]MIBG (group C), 100 MBq [^99m^Tc]TcMIBI (group D) or 100 MBq [^123^I]IPA (group E). The imaging room was conditioned to room temperature (21 ⁰C).

On day -2, 0 and 28 dynamic imaging of the rat upper torso was performed immediately after injection for 25 minutes (8x15 sec, 6x30 sec, 5x 60 sec, 3x300 sec for PET, and 15x 3 min for SPECT), while on day 30 a static image was acquired at 30–55 minutes after injection. On day -2, the rats were placed on a heating pad, while this pad was omitted on day 0, 28 and 30. Body core temperature was monitored using a rectal probe during imaging. At day 30, immediately after imaging, animals were sacrificed and relevant organs/tissues dissected, weighed and counted (automated NaI(Tl) gamma counter (Wallac Wizard).

### Image analysis (PET/SPECT)

After smoothing to 3x3x3 mm voxel size, image analysis was performed by drawing a volume of interest (VOI) around the interscapular BAT (iBAT), part of the myocardium and part of the liver (PMOD 3.0). The VOI around iBAT was drawn around the visible iBAT (on PET or SPECT image), combined with knowledge of anatomical location. Each VOI was then limited by applying a cut-off value of 30% of the (maximum–minimum) value in the VOI, thereby maintaining only tissue with true uptake. A cut-off value of 30% was found to be optimal in previous studies (unpublished), and lead to the final metabolically active volume (metabolic volume). For each VOI, the Standardized Uptake Values (SUV_mean_, Bq/cc in the region of interest, divided by the injected dose per animal weight)), as well as the metabolic volume and the total metabolic activity (SUV_mean_ multiplied by metabolic volume) were calculated. For PET, this SUV_mean_ value could be calculated directly using the output parameters from the μPET. For SPECT, the output parameter (“counts/cc”) was converted to Bq/cc using a previously determined phantom-based conversion factor of 635 (Bq per count) for both technetium-99m and iodine-123.

### Imaging (MRI/MRS)

During the MR exam the rats were placed in a cradle, which was positioned in the center of a quadrature volume coil (ø 72 mm, transmit-receive) in a 7 Tesla MR System (Bruker Biospin, Ettlingen, Germany). Rectal temperature was measured using a fiber probe. Whole-body cooling was achieved by placing of the rats on a waterbed which was connected to a heat exchanger. The water was circulated by a pump and cooled from 45 ᵒC to ~25 ᵒC, allowing rats to be cooled by three degrees Celsius from their initial rectal temperature. After this temperature loss, animals were warmed again using the waterbed until rectal temperature reached at least 36 ⁰C.

In this study we use a gradient-echo sequence with a fat suppression pulse to visualize the position of BAT. The FLASH sequence makes use of a moderate flip angle of 40 degrees, short TE of 3.9 msec and long TR of 2050 msec. When also applying a fat suppression pulse, the tissues containing relatively more fat will appear darker, allowing to precisely locate iBAT. Temperature-dependent chemical shift between H_2_O and the CH_2_ peak of fat was measured using point resolved spectroscopy (PRESS, TE = 14 ms, TR = 4500 ms, NSA = 64). Spectra were acquired from a 3x2x3 mm voxel positioned in BAT, every 4 minutes for at least 2.5 hours. Spectra were fitted using an in-house program. The chemical shift of the resonance of H_2_O and of the CH_2_ resonance of fat were determined and temperature change was quantified by assuming a temperature-dependent frequency shift of H_2_O of 0.01 ppm/⁰C.

### Western blot

All animals (control animals and cold-acclimated animals) were sacrificed at day 30 of the experiment. iBAT and visceral WAT were dissected and snap-frozen in liquid nitrogen. Western blot has been performed as stated previously [36]. In short, adipose tissue samples were incubated with RIPA buffer, frozen and after thawing lysates were passed through a 25-G needle. Lysates were seperated using SDS-PAGE prior to electrophoretic transfer onto nitrocellulose membranes. The UCP1 antibody was from Abcam (Cambridge, UK). CD36 and GLUT4 antibodies were purchased from Santa Cruz (Dallas, TX).

### Quantitative real-time PCR

All animals (control animals and cold-acclimated animals) were sacrificed at day 30 of the experiment. iBAT and visceral WAT were dissected stored in RNA-later prior to further mRNA expression analysis. Rat mRNA primer sets were developed and optimized for PPARγ, C/EBPα, Sirtuin1, UCP1, UCP2, ADRB3, DIO2, GLUT4, ATGL, LPL and PRDM16 transcripts. All gene expression data were normalized to beta-actin (see S1 Text for detailed information on mRNA analysis and S2 table for primer sequences).

### Statistics

Imaging data from animal groups were compared using ANOVA (with Bonferroni correction) for inter-group comparison, and a paired student’s t-test for intra-group comparison. mRNA data and protein data were compared using an unpaired t-test. P < 0.05 was considered to be statistically significant. All statistical tests were performed using GraphPad Prism (GraphPad Software).

## Results

### Physiological impact of the study

**Fig 2:**
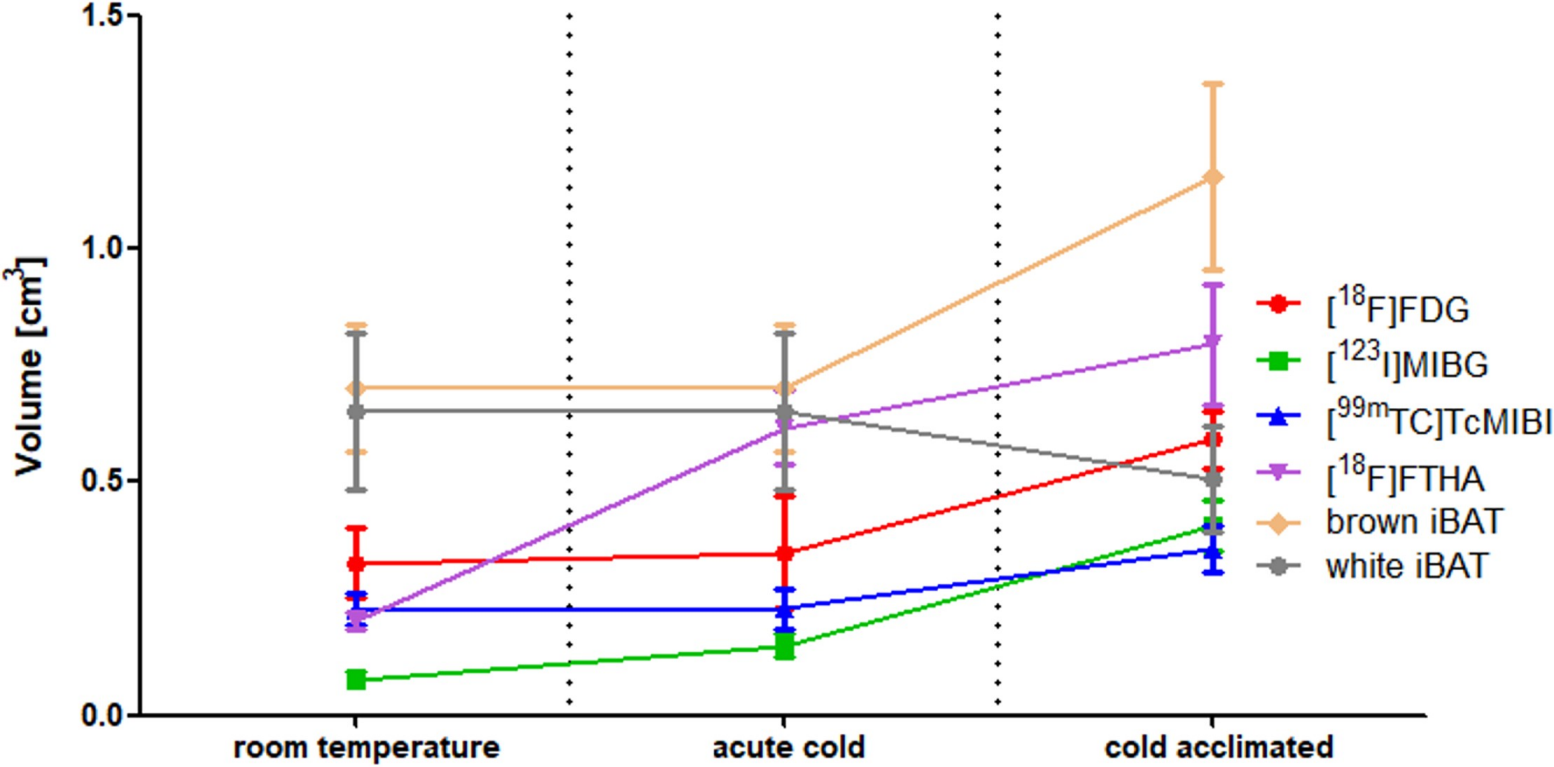
iBAT metabolic volumes of [^18^F]FDG, [^18^F]FTHA, [^123^I]MIBG and [^99m^Tc]TcMIBI, as well as iBAT and iWAT volume determined by dissection, in different temperature conditions.

Animals exposed to cold showed no signs of severe discomfort. Only during the first 2–3 days of the study the animals showed mild discomfort (shivering when exposed to cold). Animals acclimated to cold tended to gain less weight compared to animals in control conditions (1.10 ± 0.15 g/day vs 1.43 ± 0.12 g/day, p = 0.09), however the cold acclimated animals did eat more (29.4 ± 1.1 g/day vs 26.3 ± 0.9, p = 0.05). In addition, upon dissection, total interscapular adipose tissue (iAT) showed a tendency to be larger in cold-acclimated animals (1.66 ± 0.13 g vs 1.35 ± 0.08 g, p = 0.13). iAT consisted of brownish adipose tissue, covered by a layer of white-pale adipose tissue. Interscapular white adipose tissue (iWAT) reduced in weight by cold acclimation from 0.65 ± 0.08 to 0.50 ± 0.06 (p = 0.14), while iBAT increased in weight from 0.70 ± 0.07 g to 1.16 ± 0.10 g (p = 0.01) (Fig 2).

### PET and SPECT imaging

**Fig 3:**
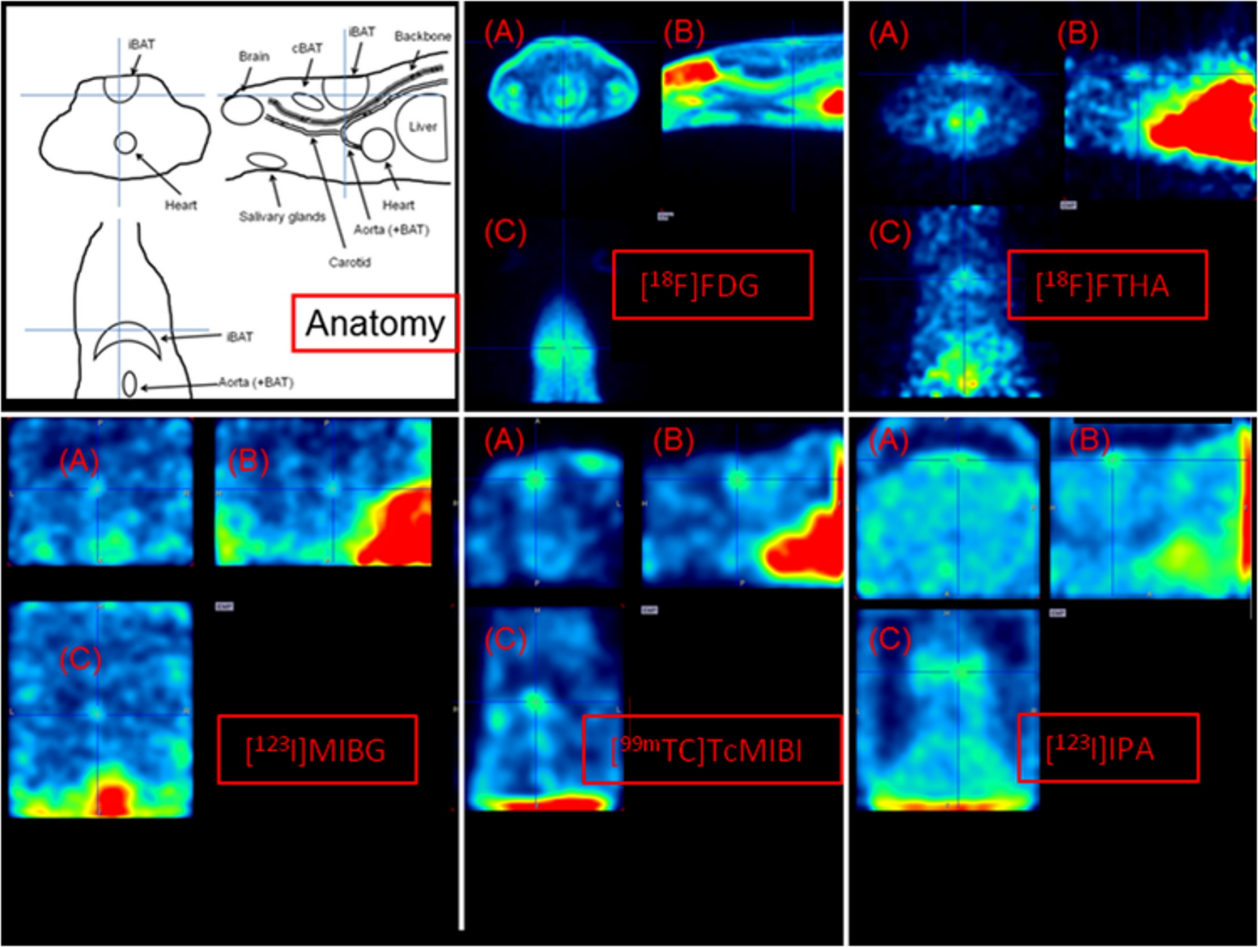
Transverse (A), sagittal (B) and coronal (C) slices of images from a room-temperature housed rat, centered on iBAT, depicting the anatomy, [^18^F]FDG distribution, [^18^F]FTHA distribution, [^123^I]MIBG distribution, [^99m^Tc]TcMIBI and [^123^I]IPA distribution. Uptake in BAT is low for all tracers.

**Fig 4:**
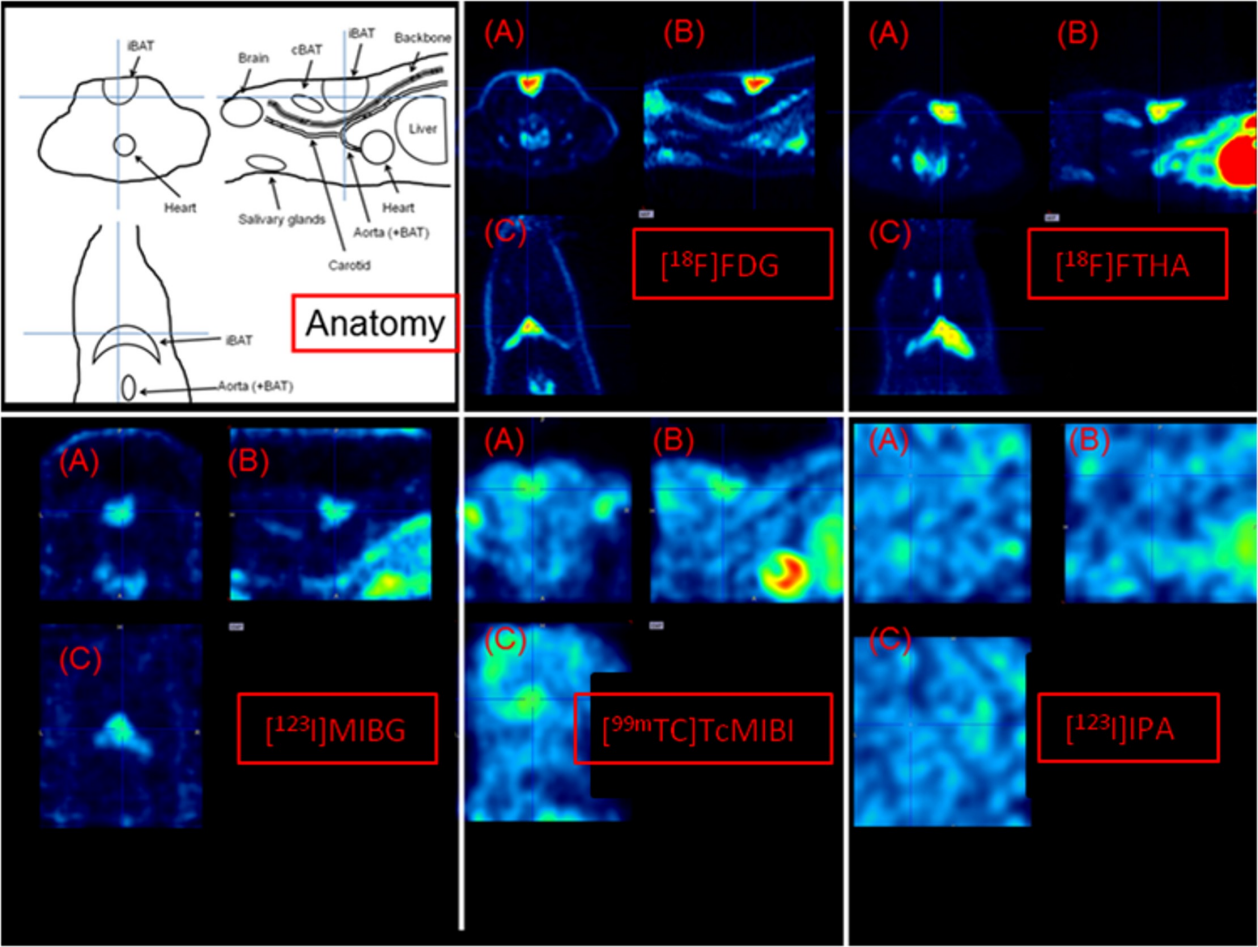
Transverse (A), sagittal (B) and coronal (C) slices of images from a cold-acclimated rat, centered on iBAT, depicting the anatomy, [^18^F]FDG distribution, [^18^F]FTHA distribution, [^123^I]MIBG distribution, [^99m^Tc]TcMIBI and [^123^I]IPA distribution. iBAT, and to some extent cBAT show a high uptake for all tracers except [^123^I]IPA.

In baseline conditions, [^18^F]FDG images showed pronounced uptake in heart and brain, and to some extent in iBAT (Fig 3). Upon acute cold exposure, uptake was still increased in the heart and the brain (although less pronounced), but now iBAT and cervical brown adipose tissue (cBAT) were also clearly visible (image not shown). After acclimation, this pattern was very well-defined (Fig 4). For [^18^F]FTHA, baseline condition images showed a high uptake in the liver and the heart, with only minor uptake in the iBAT. Upon cold exposure, uptake was clear again in the heart and the liver, however, now iBAT was also clearly visible. After acclimation, this pattern was even more pronounced, also showing cBAT (Figs 3 and 4). For [^123^I]MIBG, a pattern similar as compared to [^18^F]FTHA was observed, although uptake in BAT was less prominent (Figs 3 and 4). For [^99m^Tc]TcMIBI, baseline condition images showed again a high uptake in the liver and the heart, with only minor uptake in iBAT. Upon cold exposure or cold acclimation, uptake in iBAT increased mildly (Figs 3 and 4). Finally, [^123^I]IPA showed no specific uptake anywhere in the upper torso in any condition (Figs 3 and 4).

**Fig 5:**
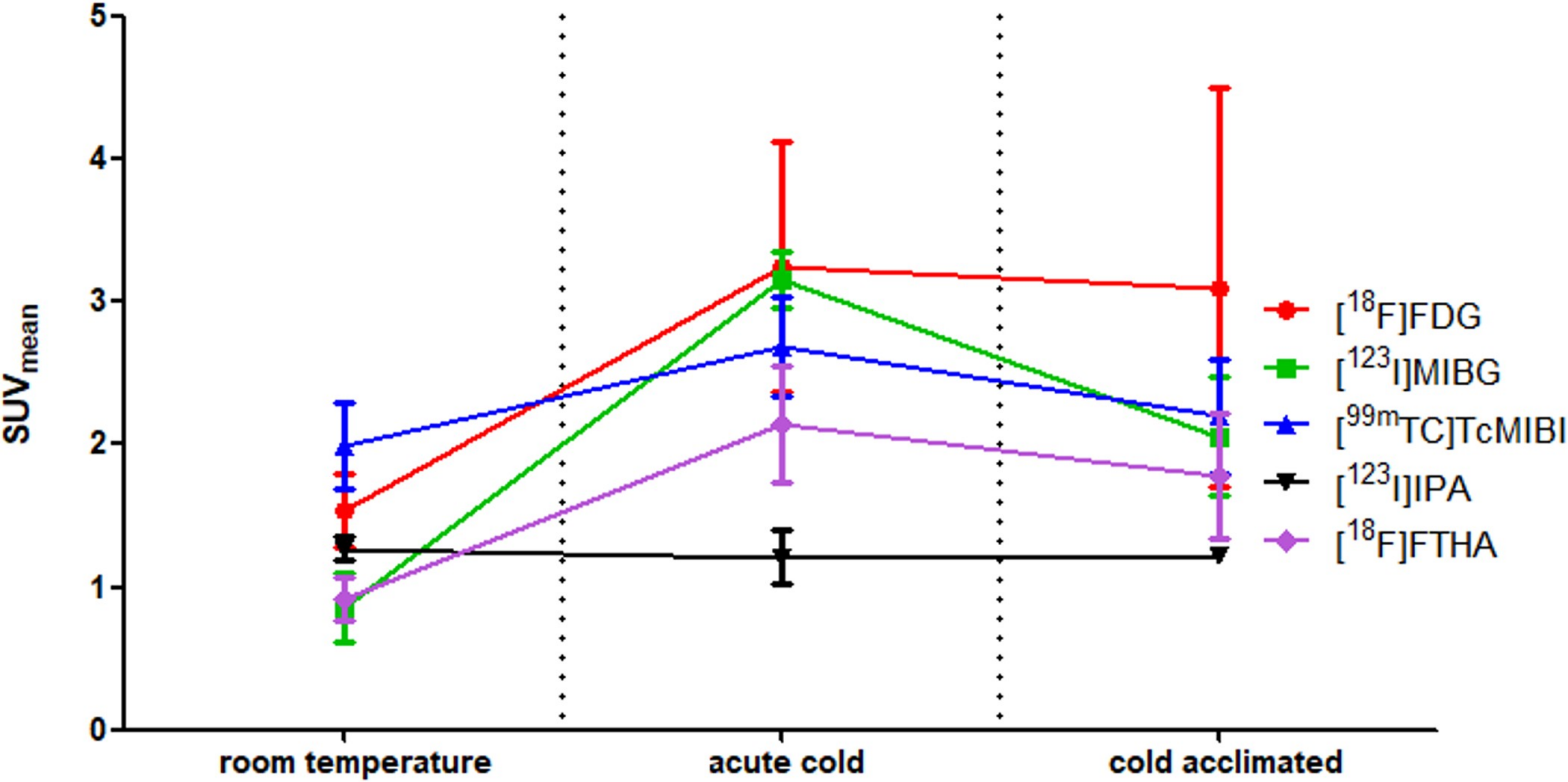
iBAT SUV_mean_ values of [^18^F]FDG, [^18^F]FTHA, [^123^I]MIBG, [^99m^Tc]MIBI and [^123^I]IPA in different temperature conditions.

Fig 5 shows the SUV_mean_ values for each tracer in iBAT in different conditions. It can be seen that acute cold exposure results in an increased SUV_mean_ value for [^18^F]FDG, [^18^F]FTHA, [^123^I]MIBG and [^99m^Tc]TcMIBI in iBAT when compared to baseline conditions (p-values are respectively 0.11; 0.03; 0.001 and 0.02) (also see Table 1). Cold acclimation results in similar increases when compared to room temperature values. As shown in Fig 2, the metabolically active tissue volume in iBAT is increased after cold acclimation when investigated with [^18^F]FDG, [^18^F]FTHA, [^123^I]MIBG and [^99m^Tc]TcMIBI. Surprisingly, this increase in labeled tissue is already present after single acute cold exposure for [^18^F]FTHA and to some extent also [^123^I]MIBG. [^123^I]IPA shows poor uptake in iBAT, preventing accurate VOI drawing. As a result, it is only possible to estimate SUV_mean_ [^123^I]IPA values in iBAT, but not the metabolic volume or total metabolic activity. The SUV_mean_ values show no change over the course over the experiment.

**Table 1:** SUV, metabolic volume and total metabolic activity of [^18^F]FDG, [^18^F]FTHA, [^123^I]MIBG, [^99m^Tc]TcMIBI and [^123^I]IPA in iBAT in baseline conditions, after acute cold exposure, after cold acclimation and after uptake in the cold. ↑ indicates a p-value < 0,10 when compared to baseline conditions, while † indicates a p-value < 0,10 when compared to the value after acute cold exposure. NQ indicates “not quantifiable”, while NA means these data are not available.

iBAT	Baseline	Acute cold exposure	Acclimated to cold	Uptake in cold
Tracer	SUV (g/cm^3^)			
[^18^F]FDG	1.54±0.26	3.24±0.88	3.10±1.39	3.23±1.51
[^18^F]FTHA	0.92±0.07	2.14±0.20↑	1.78±0.22↑	2.36±0.24↑
[^123^I]MIBG	0.86±0.24	3.15±0.20↑	2.06±0.42↑	1.82±0.12↑
[^99m^Tc]TcMIBI	1.99±0.31	2.69±0.34↑	2.20±0.40	2.83±0.56
[^123^I]IPA	1.27±0.04	1.21±0.09	1.22±0.01	NA
	Metabolic volume (cm^3^)			
[^18^F]FDG	0.32±0.07	0.35±0.12	0.59±0.06↑	0.54±0.03↑
[^18^F]FTHA	0.20±0.01	0.62±0.04↑	0.80±0.06↑,†	0.64±0.12↑
[^123^I]MIBG	0.08±0.02	0.15±0.02↑	0.41±0.05↑,†	0.30±0.03↑,†
[^99m^Tc]TcMIBI	0.23±0.03	0.23±0.05	0.36±0.05↑,†	0.42±0.05↑,†
[^123^I]IPA	NQ	NQ	NQ	NA
	Total metabolic activity (SUV x Volume) (cm^3^)			
[^18^F]FDG	0.48±0.09	0.89±0.11↑	1.71±0.52↑	1.78±0.66↑
[^18^F]FTHA	0.18±0.01	1.41±0.16↑	1.41±0.17↑	1.54±0.38↑
[^123^I]MIBG	0.07±0.04	0.46±0.06↑	0.79±0.12↑,†	0.54±0.02↑,†
[^99m^Tc]TcMIBI	0.43±0.06	0.59±0.08	0.74±0.06↑	1.25±0.34↑,†
[^123^I]IPA	NQ	NQ	NQ	NA

In comparison, the myocardium and the liver showed no significant changes in SUV_mean_ values throughout the study for any of the tracers, nor did their (metabolic) volume change.

In addition, there was no difference in data obtained from images that were acquired by injecting the tracer in cold-acclimated animals under the camera, in a room where the animal was no longer exposed to cold during tracer distribution, and images that were acquired after the tracer had been given the chance to distribute in the cold-acclimated animal for 30 minutes in a cold environment (also see Table 1).

Analysis of biodistribution data obtained from dissected tissue (Table 2) confirms the data obtained from images. Dissection data did allow quantification of a number of aspects that were impossible to derive from imaging data, such as the discrimination between iWAT and iBAT, thereby clearly showing that only iBAT has tracer uptake. Perivascular BAT, often visible but never quantifiable on images due to its small size and structure, showed a similar uptake to that in iBAT when corrected for weight.

**Table 2:** Biodistribution (determined by dissection) of [^18^F]FDG, [^18^F]FTHA, [^123^I]MIBG, [^99m^Tc]TcMIBI and [^123^I]IPA in selected organs 1h after tracer uptake in the cold.

%ID/g	[^18^F]FDG	[^18^F]FTHA	[^123^I]MIBG	[^99m^Tc]TcMIBI	[^123^I]IPA
iBAT	2.89±0.66	1.36±0.39	0.93±0.23	0.90±0.10	0.37±0.04
iWAT	0.41±0.12	0.16±0.06	0.18±0.03	0.16±0.02	0.26±0.03
Perivascular BAT	3.65±1.55	1.08±0.30	2.02±0.54	1.49±0.37	0.52±0.05
Intestinal WAT	0.19±0.01	0.14±0.05	0.12±0.03	0.05±0.01	0.22±0.10
Subcutaneous WAT	0.18±0.02	0.08±0.01	0.17±0.05	0.10±0.03	0.04±0.07
Liver	0.37±0.04	3.16±0.38	1.64±0.14	1.25±0.26	0.68±0.04
Lung	0.36±0.05	0.53±0.11	3.45±0.39	0.55±0.02	0.57±0.02
Heart	2.34±0.25	1.01±0.08	4.35±0.34	3.00±0.23	0.58±0.01
Muscle	0.10±0.01	0.07±0.01	0.11±0.01	0.12±0.02	0.40±0.04
Salivary glands	0.51±0.05	0.43±0.06	1.87±0.28	1.93±0.30	0.34±0.23
Blood	0.34±0.06	0.12±0.01	0.16±0.01	0.02±0.01	0.93±0.02

### MR Spectroscopy

**Fig 6:**
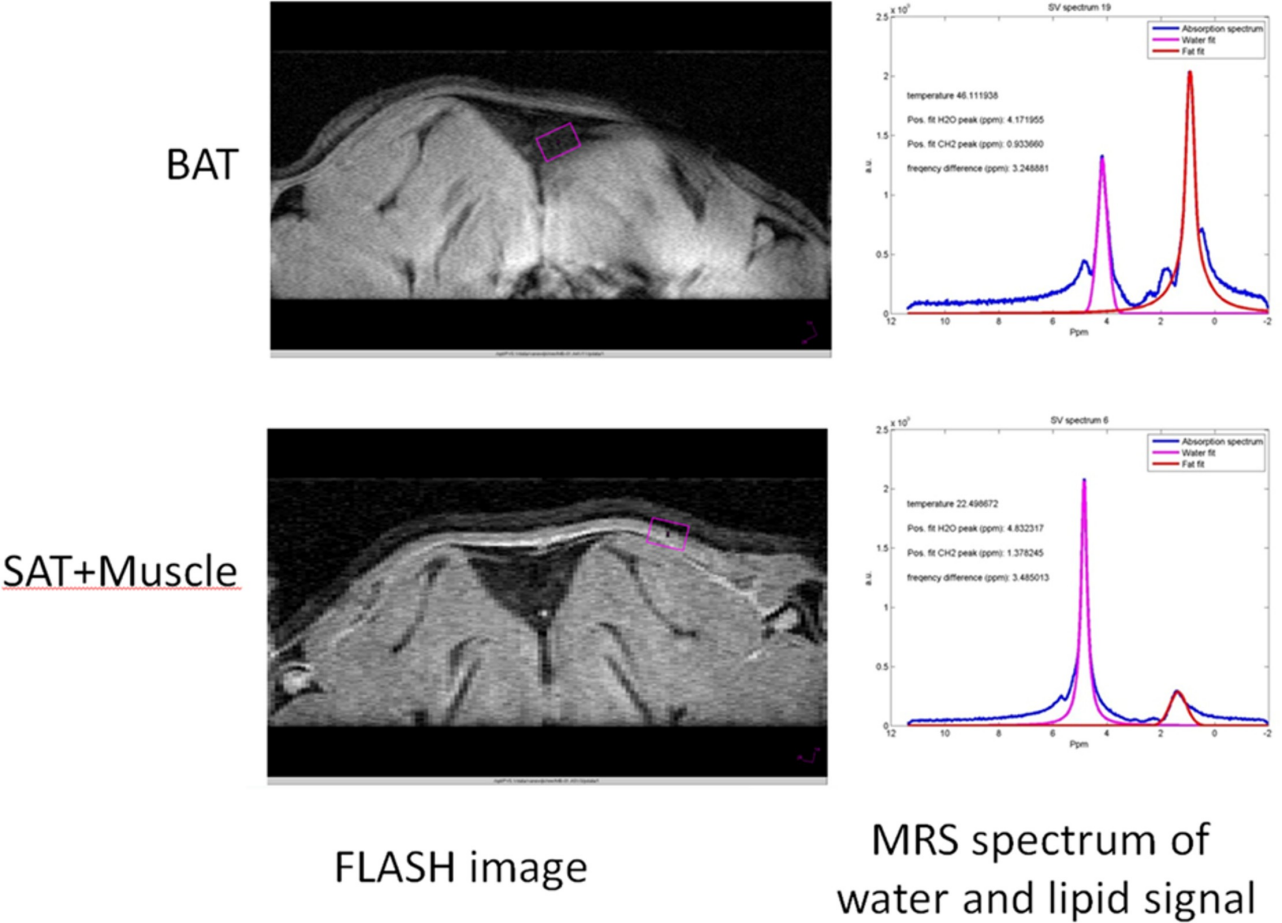
MRI Images and their corresponding spectra from different positions in the body. BAT = Brown Adipose tissue, SAT = Subcutaneous Adipose Tissue.

The use of the gradient-echo sequence with a fat suppression pulse made it possible to visualize the position of BAT in rat (Fig 6). The FLASH sequence combined with a fat suppression pulse, makes the WAT darker in comparison to the BAT. Temperature was determined in BAT, and as a reference, also in muscle tissue by MRS and furthermore, rectal temperature was determined. The one-hour cooling protocol resulted in a decreased temperature of 1.9 ± 0.9 ⁰C in non-acclimated animals and a decrease of 1.8 ± 0.9 ⁰C in cold-acclimated animals (also see Table 3). During this period of cooling, MRS-derived temperature in BAT decreased by 1.2 ± 0.9 ⁰C in non-acclimated animals but increased by 0.3 ± 1.5 ⁰C in cold-acclimated animals (p = 0.22). MRS-derived temperature in muscular tissue (including a minor amount of subcutaneous adipose tissue) decreased by 2.4 ± 1.5 ⁰C in non-acclimated animals and by 1.4 ± 1.8 ⁰C in cold-acclimated animals (p = 0.18). In cold-acclimated animals the decrease in BAT temperature is less pronounced than that in subcutaneous muscular tissue (p = 0.08) or in the rectum (p = 0.004), while temperature in non-acclimated animals showed a similar time course in BAT vs. muscle and rectal temperature (p > 0.15 and > 0.17 respectively).

**Table 3:** MRS-derived temperature differences in BAT and Muscle and rectal temperature difference in control and cold-acclimated animals. There is no statistically significant difference between cold-acclimated and control animals.

Temperature change upon 1 hour of cooling	Control	Cold-acclimated
Rectal	-1.9 ± 0.9°C	-1.8 ± 0.9°C
MRS-derived muscle	-2.4 ± 1.5°C	-1.4 ± 1.8°C
MRS-derived BAT	-1.2 ± 0.9°C	+0.3 ± 1.5°C

### mRNA expression and protein expression

mRNA expression of genes related to BAT metabolism (PPARγ2, C/EBPα, UCP1, ADRB3, DIO2, ATGL, PRDM16) were higher in iBAT compared to iWAT. Similar results were observed for GLUT4 mRNA expression when comparing iBAT to iWAT although the results did not reach statistical significance (data not shown). When examining protein expression, control animals showed higher levels of UCP1 and GLUT4 protein expression in iBAT compared to WAT, however CD36 protein expression was similar between iBAT and WAT (p = 0.43).

Cold acclimation did not alter mRNA expression of the selected genes in BAT. PRDM16 mRNA expression decreased following cold acclimation in BAT (p = 0.013) (S1 Table). No changes in protein levels of UCP1, GLUT4 or CD36 in BAT were detected following cold acclimation using western blot (S3 Table).

## Discussion

In this study, we used a rat model to assess BAT molecular imaging with [^18^F]FDG, [^18^F]FTHA, [^123^I]MIBG, [^99m^Tc]TcMIBI and [^123^I]IPA in baseline and different cold-stimulated conditions. Additionally we investigated temperature of BAT and muscle as a negative control in cold stimulated conditions by MRS and analyzed mRNA and protein expression of a number of relevant genes.

In our model, cold acclimation caused a clear growth of iBAT volume, while white interscapular adipose tissue decreased in volume. mRNA and protein levels easily allowed to distinguish brown from white adipose tissue in our study, but did not show any significant differences between control and cold-acclimated animals. Unlike reported in literature [28, 37–39] we cannot report an up regulation of BAT specific genes (e.g. UCP1, PPARγ, LPL) (S1 Table). This can be caused by several reasons e.g. the biological half-life of certain mRNAs is in the order of hours and therefore dependent on diurnal variations. Timing of sampling might therefore of high importance [40, 41]. Furthermore, the choice of housekeeping genes is a topic of discussion. In our experiment, a broadly used housekeeping gene, GAPDH was variable throughout the experiment and therefore not suitable as reference. As we expose the animals to cold over a long period, cell type alterations in the whole fat depot are possible which can be caused e.g. by cell differentiation. In such experiments, the choice of housekeeping gene is especially difficult, as these alterations might affect the transcriptional apparatus and by that the absolute expression of the housekeeping gene might be changed [28].

Protein expression levels of relevant genes did allow, just like mRNA expression levels, to distinguish BAT from WAT. However, just like mRNA expression levels, cold-acclimation did not lead to a significant change in protein expression levels (S3 Table). Additionally, to the mentioned problems above, it might be possible that 6 h of cold exposure per day might not be sufficient to observe an upregulation of mRNA or proteins. Due to ethical restrictions it was not possible to extend exposure times or even house the rats at 6°C.

Although we were not able to report any differences in mRNA/protein levels of BAT in cold acclimated vs. control animals as reported in literature (e.g. UCP1 [28], PPARγ [37], CD36 [38, 39]) we were able to distinguish between BAT and WAT by these techniques, which was important for *ex vivo* analysis of tissue samples.

MRS showed that the temperature drop in BAT was less pronounced after cold acclimation, which can be interpreted as an increase in BAT activity. In cold acclimated animals BAT temperature differed significantly in comparison to rectal and muscle temperature. A comparison between BAT temperatures of non cold acclimated vs. cold acclimated animals showed a clear trend towards higher temperatures in cold acclimated animals but did not reach significant differences. This is also in accordance with the results obtained from the biodistribution where cold acclimation significantly increased iBAT volume. A larger BAT depot, which results from cold acclimation, would be better suited to maintain or even increase its temperature during cold due to more active BAT.

[^18^F]FDG allowed iBAT visualization in warm conditions as well as in animals exposed to acute cold and cold-acclimated animals. If animals were exposed to cold, cBAT was visible as well. [^18^F]FTHA, [^123^I]MIBG and [^99m^Tc]TcMIBI poorly visualized iBAT in warm conditions, but clearly showed iBAT and to some extent also cBAT in animals exposed to acute cold and when animals were acclimated to cold. [^123^I]IPA did not visualize iBAT in any setting and is therefore not suited as a BAT imaging tracer.

The analysis of tracer SUV_mean_ values in iBAT showed that, when compared to warm conditions, the focal uptake of [^18^F]FDG, [^18^F]FTHA and [^123^I]MIBG in animals exposed to acute cold was about double, while for [^99m^Tc]TcMIBI the increase was by a factor of 1.3. Considering the time window of 4 hours cold exposure, the factor of 1.3 is probably due to an increase in perfusion / blood flow and not to an increase in mitochondrial density. This confirms earlier findings of increased blood flow due to cold exposure with other tracers such as [^201^Tl]thallium chloride [12].

Cold-acclimation did not further increase focal uptake of any of the tracers, indicating that one single 4 h cold exposure is sufficient to reach a maximum tracer uptake in (the metabolically active part of) iBAT in our rodent model. This is in contrast to what has been reported in humans, where the SUV_mean_ of active BAT continued to increase upon chronic cold acclimation [29, 42]. We hypothesized that this effect is induced by the fact that room temperature (22°C) is already below the thermoneutral zone of rats (~30°C) [43] so that a slight cold acclimation already takes place under control conditions.

The volume of tissue with a high tracer uptake shows a different pattern, as the volume after cold exposure is similar to that in warm conditions and is only increased upon cold-acclimation for [^18^F]FDG and [^99m^Tc]MIBI. For [^18^F]FTHA and [^123^I]MIBG the increase in volume already starts after acute cold exposure, and then increases further upon cold-acclimation. These findings are to some extent not congruent with those found in literature from clinical studies, in which [^18^F]FTHA showed a lack of uptake under acute cold exposure and less uptake compared to FDG upon cold stimulation [15]. This difference in [^18^F]FTHA may be due to a metabolic difference in animal species (rat vs. human), but may also be due to the higher difficulty of delineating brown adipose tissue in humans compared to rats. The volume increase of active BAT is in accordance with the results obtained from MRS and the biodistribution where increased BAT temperature or volume could only be found after cold acclimation.

The total BAT activity is increased firstly by an acute cold exposure which induced an increase in metabolic activity in a small volume of iBAT (i.e. activation of existing brown adipocytes), and secondly by a cold-acclimation induced increased in metabolically active volume (i.e. increased number of activated brown adipocytes). This is different from humans, where cold acclimation leads to both an increased volume and increased SUV_mean_ value [29]. It is possible that, compared to the necessary presence of frequently activated BAT in rodents, human BAT may be more facultative and therefore can be stimulated to a greater extent when compared to baseline room temperature conditions. Both methods, dissection of iAT and volume quantification by tracer uptake could show an increase in BAT volume due to cold acclimation. It is important to mention that BAT volume determined by dissection always shows higher values as determined by tracer uptake mainly due to the fact that PET/SPECT data only show active parts of BAT.

All tracer except for [^123^I]IPA were able to visualize BAT under acute and acclimated cold conditions. With the support of the different tracer we were able to investigate different aspects of BAT metabolism and features during cold exposure and compare these results to other measurement techniques. We were able to show BATs dependence on glucose and fatty acid metabolism by [^18^F]FDG and [^18^F]FTHA by PET and uptake was increased due to acute cold exposure. With [^99m^Tc]MIBI we were able to visualize BATs perfusion and with [^123^I]MIBG expression of sympathetic nerve endings which are responsible for norepinephrine release and therefore for BAT activation. Both tracers could show increased uptake after acute cold exposure. Only [^123^I]IPA was not able to visualize BAT under all temperature conditions, indicating an independence of BAT on LAT1-4 amino acid transport system. Invasive measurements of BAT volume were coherent with results obtained by MRS and dissection of iBAT.

The current imaging procedures in humans, with different cooling protocols and different tracers in different centers, is known to confer significantly different results in terms of total BAT activity, especially when comparing different tracers [1, 44–48]. Our data may offer an explanation for this phenomenon, as tracer uptake can be dependent on the amount of cold exposure, and the nature of this dependence seems different for each tracer.

## Conclusion

[^18^F]FDG, [^18^F]FTHA, [^123^I]MIBG and [^99m^Tc]TcMIBI, but not [^123^I]IPA, are suitable for imaging aspects of BAT activity using non-invasive molecular imaging, while MRS is able to quantify heat production in BAT, even in an animal model where classical ex vivo techniques fail to show significant trends. The uptake in BAT of each tracer responds differently to acute cold exposure, and cold-acclimation does not increase BAT tracer uptake as strongly in our animal model as it does in humans. Those differences in tracer uptake characteristics should be considered during study planning as well as it should be translated to clinical applications.

## Supporting information

S1 Table mRNA expression levels. Relative mRNA expression levels (shown as a percentage of beta-actin expression) of different genes in iBAT, iWAT and ipWAT. Cold-acclimation = animals exposed to cold for 6h per day for 4 weeks.–indicates a value below 1%. (*) indicates a statistically significant difference between animals housed at RT or in cold-acclimated conditions, while (†) indicates a statistically significant difference between brown adipose tissue and white adipose tissue.
(DOC)

S2 Table Primer sequences for RT-PCR. For each gene, a minimum of 20 bases was chosen for the primer length, which was increased where necessary to ensure specificity.
(DOC)

S3 Table Protein expression. Relative Protein expression [AU] in iBAT and visceral WAT after dissection and workup. Cold-acclimation = animals exposed to cold for 6h per day for 4 weeks. (†) indicates a statistically significant difference between brown adipose tissue and white adipose tissue.
(DOC)

S1 Dataset Raw data microPET image analysis (excel). (XLS)

S2 Dataset Raw data MRS analysis (excel). (XLSX)

S1 Text mRNA quantification. (DOC)

S2 Text NC3Rs ARRIVE guidelines checklist. (PDF)
