# Supplementary material for: Characterization of BAT activity in rats using invasive and non-invasive techniques
Source: PLoS One. 2019 May 15;14(5):e0215852. doi: 10.1371/journal.pone.0215852 (PMC6519816; doi:10.1371/journal.pone.0215852)
Supplement: S1 Table — Relative mRNA expression levels (shown as a percentage of beta-actin expression) of different genes in iBAT, iWAT and ipWAT. Cold-acclimation = animals exposed to cold for 6h per day for 4 weeks.–indicates a value below 1%. (*) indicates a statistically significant difference between animals housed at RT or in cold-acclimated conditions, while (†) indicates a statistically significant difference between brown adipose tissue and white adipose tissue. (DOC) [file pone.0215852.s001.doc]

**Supplemental information (S1 Table)**

**S1 Table** : Supplemental Table (1): Relative mRNA expression levels (shown as a percentage of beta-actin expression) of different genes in iBAT, iWAT and ipWAT. Cold-acclimation = animals exposed to cold for 6h per day for 4 weeks. – indicates a value below 1%. (*) indicates a statistically significant difference between animals housed at RT or in cold-acclimated conditions, while (†) indicates a statistically significant difference between brown adipose tissue and white adipose tissue.

|  | Room temperature housing | | | Cold acclimation | | |
| --- | --- | --- | --- | --- | --- | --- |
| mRNA gene | iBAT | iWAT | ipWAT | iBAT | iWAT | ipWAT |
| PPARγ2 (†) | 139±25 | 35±12 | 50±7 | 111±11 | 32±12 | 39±6 |
| C/EBPα (†) | 237±43 | 77±24 | 93±20 | 110±28 (*) | 55±19 | 80±22 |
| FOXo1 | 139±38 | 123±7 | 71±19 | 144±20 | 84±21 | 85±10 |
| Sirtuin1 | 198±31 | 145±15 | 125±24 | 139±27 | 96±2 | 84±8 |
| UCP1 (†) | 238±43 | 77±24 | 93±20 | 110±28 (*) | 55±19 | 80±22 |
| UCP2 | 4±3 | 13±8 | 10±2 | 6±1 | 14±4 | 20±9 |
| ADRB3 (†) | 517±175 | 124±77 | 157±63 | 193±64(*) | 50±30 | 25±18(*) |
| DIO2 (†) | 5±3 | - | - | 6±1 | - | - |
| GLUT4 (†) | 29±5 | 3±1 | 2±1 | 24±5 | 4±1 | 6±1 |
| ATGL (†) | 295±78 | 66±55 | 51±21 | 183±48 | 20±11 | 17±5 |
| LPL | 120±46 | 83±38 | 136±20 | 129±18 | 86±41 | 130±30 |
| PRDM16 (†) | 125±15 | 11±4 | 7±1 | 55±3(*) | 4±1 | 8±5 |
