# Supplementary material for: Characterization of BAT activity in rats using invasive and non-invasive techniques
Source: PLoS One. 2019 May 15;14(5):e0215852. doi: 10.1371/journal.pone.0215852 (PMC6519816; doi:10.1371/journal.pone.0215852)
Supplement: S2 Table — For each gene, a minimum of 20 bases was chosen for the primer length, which was increased where necessary to ensure specificity. (DOC) [file pone.0215852.s002.doc]

**Supplemental information (S2 Table)**

**S2 Table** Supplemental Table (2): Primer sequences for RT-PCR. For each gene, a minimum of 20 bases was chosen for the primer length, which was increased where necessary to ensure specificity.

| mRNA gene | Forward | Reverse |
| --- | --- | --- |
| PPARγ2 | GCCTGCGGAAGCCCTTTGGT | CAGCAAGCCTGGGCGGTCTC |
| C/EBPα | GGTACGGCGGGAACGCAACA | GAAGATGCCCCGCAGCGTGT |
| FOXo1 | GGACAGCCGCGCAAGACCAG | TTGAATTCTTCCAGCCCGCCGA |
| Sirtuin1 | GCTCGCCTTGCGGTGGACTT | GACGGCTGGAACTGTCCGGG |
| UCP1 | ACCTTCCCGCTGGACACTGC | GCCAGGGTGGTGATGGTCCC |
| UCP2 | GCTGGGACAGCTGCCTGCAT | CGGTGCGCACTAGCCCTTGA |
| ADRB3 | ACGAGATGGCTCCGTGGCCT | CAGCAGGTTGCCTCCCACCG |
| DIO2 | GGGCTGCGCTGTGTCTGGAA | CGGCCCCATCAGCGGTCTTC |
| GLUT4 | ACCCACCGGCAGCCTCTGAT | TAGGCTGGCTGTCCCACCCC |
| ATGL | TGGACGCCTGGGCATCTCCC | TGTGCTGCAGACATTGGCCTGG |
| LPL | AGTCTGGCTGACACTGGACAAACA | CACCCTGGGTTAGCCACCGTTTA |
| PRDM16 | CCAATCAGGCGGGGTCTGGC | GCACCAACAGTTCCTCTCCAGGC |
| Beta-actin | CGACAACGGCTCCGGCATGT | TAGGGCGGCCCACGATGGAG |
