# Supplementary material for: Characterization of BAT activity in rats using invasive and non-invasive techniques
Source: PLoS One. 2019 May 15;14(5):e0215852. doi: 10.1371/journal.pone.0215852 (PMC6519816; doi:10.1371/journal.pone.0215852)
Supplement: S3 Table — Relative Protein expression [AU] in iBAT and visceral WAT after dissection and workup. Cold-acclimation = animals exposed to cold for 6h per day for 4 weeks. (†) indicates a statistically significant difference between brown adipose tissue and white adipose tissue. (DOC) [file pone.0215852.s003.doc]

**Supplemental information (S3 Table)**

**S3 Table** : Supplemental Table (3): Relative Protein expression [AU] in iBAT and visceral WAT after dissection and workup. Cold-acclimation = animals exposed to cold for 6h per day for 4 weeks. (†) indicates a statistically significant difference between brown adipose tissue and white adipose tissue.

|  | Room temperature housing | | Cold acclimation | |
| --- | --- | --- | --- | --- |
| Protein | iBAT | visceral WAT | iBAT | visceral WAT |
| UCP1 | 47697±23433 | - | 71830±68745 | - |
| CD36 (†) | 2585±1241 | 1082±363 | 2080±1288 | 901±474 |
| GLUT4 (†) | 21829±3905 | 8882±8676 | 22075±17833 | 5506±1856 |
