# Supplementary material for: Characterization of BAT activity in rats using invasive and non-invasive techniques
Source: PLoS One. 2019 May 15;14(5):e0215852. doi: 10.1371/journal.pone.0215852 (PMC6519816; doi:10.1371/journal.pone.0215852)
Supplement: S1 Text — (DOC) [file pone.0215852.s006.doc]

**Supplemental information (S1)**

**S1 Text: mRNA quantification**

Selected tissues were harvested from 16-week old Wistar rats that were housed continuously at room temperature (n=3) and frozen in RNA-later (Qiagen) at -80 ⁰C for later RNA isolation and gene expression analyses. The selected tissues were brown interscapular adipose tissue (iBAT), white interscapular adipose tissue (iWAT) and intraperitoneal WAT (ipWAT). Rat mRNA primer sets were PPARγ, C/EBPα,, Sirtuin1, UCP1, UCP2, ADRB3, DIO2, GLUT4, ATGL, LPL and PRDM16.

Tissues were lysed, disrupted and homogenized using the Mini-BeadBeater by violently agitating 2 ml screw-cap microvials containing small glass beads and RLT buffer (Qiagen) supplemented with 1% 2-mercaptoethanol (Sigma). Total cellular RNA was isolated from lysed cells using RNeasy Mini kit (Qiagen) according to manufacturer’s instructions. Genomic DNA was removed by DNAse I (Invitrogen) treatment followed by reverse transcription using random hexamer primers (Invitrogen) and Superscript III Reverse Transcriptase (Invitrogen) according to standard procedures. Primers for quantitative PCR analysis were generated using primer-BLAST software (NCBI). All primers were selected to span exon-exon boundaries with a maximal amplification length of 300 bp. Primer specificity was confirmed by melting curve analyses and product size confirmation by gel-based PCR analyses. Real-time PCR was performed with Sybr green detection (SensiMix SYBR & Fluorescein Kit, Bioline) using an iCycler iQ (Biorad) and primers (10pmol) specific for the different genes. The PCR program consisted of 10 min. initial heating at 95°C (*hot start* polymerase), followed by 35 cycles amplification (30 sec. at 95°C, 20 sec. at the optimized annealing temperature and 20 sec. at 72°C) and a final heating up to 90°C (increasing 0.5°C/ 30 sec.) for the generation of a melting curve. Quantification of a gene of interest was done by generating a standard curve using serial dilutions of a reference sample (mixed cDNA of BAT of 3 different rats). Relative expression levels were obtained by normalizing to the expression of β-actin in the corresponding sample. Each data point of the graphs was generated by determining expression of the gene of interest on the different rats in duplicate and normalizing these data to the corresponding expression of β-actin used as housekeeping gene in our assay (β-actin was chosen as it was the most stable housekeeping gene in our experiment).
